# Dysregulation of Macrophage-Secreted Cathepsin B Contributes to HIV-1-Linked Neuronal Apoptosis

**DOI:** 10.1371/journal.pone.0036571

**Published:** 2012-05-31

**Authors:** Eillen J. Rodriguez-Franco, Yisel M. Cantres-Rosario, Marines Plaud-Valentin, Rafael Romeu, Yolanda Rodríguez, Richard Skolasky, Viviana Meléndez, Carmen L. Cadilla, Loyda M. Melendez

**Affiliations:** 1 Department of Microbiology and Medical Zoology, University of Puerto Rico Medical Sciences Campus, San Juan, Puerto Rico, United States of America; 2 Department of Biochemistry, University of Puerto Rico Medical Sciences Campus, San Juan, Puerto Rico, United States of America; 3 Department of Biology, University of Puerto Rico, Rio Piedras Campus, San Juan, Puerto Rico, United States of America; 4 Department of Biotechnology, University of Puerto Rico, University of Puerto Rico, Mayaguez Campus, Mayaguez, Puerto Rico, United States of America; 5 Department of Orthopedic Surgery, John Hopkins University; Baltimore, Maryland, United States of America; University of Montreal, Canada

## Abstract

Chronic HIV infection leads to the development of cognitive impairments, designated as HIV-associated neurocognitive disorders (HAND). The secretion of soluble neurotoxic factors by HIV-infected macrophages plays a central role in the neuronal dysfunction and cell death associated with HAND. One potentially neurotoxic protein secreted by HIV-1 infected macrophages is cathepsin B. To explore the potential role of cathepsin B in neuronal cell death after HIV infection, we cultured HIV-1_ADA_ infected human monocyte-derived macrophages (MDM) and assayed them for expression and activity of cathepsin B and its inhibitors, cystatins B and C. The neurotoxic activity of the secreted cathepsin B was determined by incubating cells from the neuronal cell line SK-N-SH with MDM conditioned media (MCM) from HIV-1 infected cultures. We found that HIV-1 infected MDM secreted significantly higher levels of cathepsin B than did uninfected cells. Moreover, the activity of secreted cathepsin B was significantly increased in HIV-infected MDM at the peak of viral production. Incubation of neuronal cells with supernatants from HIV-infected MDM resulted in a significant increase in the numbers of apoptotic neurons, and this increase was reversed by the addition of either the cathepsin B inhibitor CA-074 or a monoclonal antibody to cathepsin B. *In situ* proximity ligation assays indicated that the increased neurotoxic activity of the cathepsin B secreted by HIV-infected MDM resulted from decreased interactions between the enzyme and its inhibitors, cystatins B and C. Furthermore, preliminary *in vivo* studies of human post-mortem brain tissue suggested an upregulation of cathepsin B immunoreactivity in the hippocampus and basal ganglia in individuals with HAND. Our results demonstrate that HIV-1 infection upregulates cathepsin B in macrophages, increases cathepsin B activity, and reduces cystatin-cathepsin interactions, contributing to neuronal apoptosis. These findings provide new evidence for the role of cathepsin B in neuronal cell death induced by HIV-infected macrophages.

## Introduction

HIV-1 infects brain mononuclear phagocytes (MP; monocytes, perivascular macrophages, dendritic cells and microglia) leading to a chronic viral infection and consequent neurological impairments, designated as HIV-associated neurocognitive disorders (HAND) [1]. Importantly, the prevalence of HAND remains high despite the widespread use of combination antiretroviral therapy (cART), and affects 30–50% of infected individuals [2], [3], [4]. Viral invasion of the central nervous system (CNS) occurs as a consequence of blood-derived monocytes entering the brain across the blood brain barrier (BBB) [5], [6], [7]. Although HIV-1 penetrates the CNS soon after viral infection, neurological symptoms occur only after immune suppression and coincide with the development of AIDS [8]. What underlies disease is the secretion of soluble viral and cellular neurotoxins from activated and infected perivascular macrophages and microglia [9], [10]. The secretion of these factors, together with severe dysregulation of macrophage function, can lead to neuronal dysfunction and apoptosis [11], [12], resulting in cognitive impairment.

Although cART can restore immune function by suppressing viral replication and decreasing the inflammatory neurotoxins that exacerbate the signs and symptoms of HAND [13], it cannot prevent disease progression [14], [15]. This failure may result from limited drug penetrance into the CNS, viral mutations, and/or inadequate therapy compliance [16], [17]. Among the cellular proteins that could promote neuronal apoptosis, if not properly regulated, is cathepsin B, a cysteine protease of lysosomal origin involved in various important cellular processes such as antigen processing and presentation [18], apoptosis [19], inflammation and neurodegeneration [20]. Cathepsin B is found in high abundance in activated macrophages and has been shown to be involved in programmed cell death [21]. Under normal conditions cathepsin B is under stringent regulation due to its potential detrimental effects on cells. However, oxidative stress and soluble cytokines may promote the release of cathepsin B from lysosomes and extracellular secretion by MP. Therefore cathepsin B could in turn contribute to the apoptosis of adjacent cells by promoting mitochondrial release of cytochrome c [21].

How HIV-1 infection of macrophages affects interactions between cathepsin B and its inhibitors, cystatins B and C, and thereby potentially impact neuronal survival was assessed in the current study. Human monocyte-derived macrophages (MDM) were cultured and infected with HIV-1_ADA_ for 12 days, and the expression of intracellular and extracellular cathepsin B, cystatin B, and cystatin C in uninfected and HIV-1 infected cells was monitored over time. Our results demonstrate that HIV-1 infection of MDM leads to increased cathepsin B RNA levels, and increased cathepsin B secretion, activity, and neurotoxicity. We also show that cathepsin B is released outside of the lysosome after HIV infection and that its interactions with cystatins B and C are decreased. Thus, HIV infection alters cathepsin B activity and secretion by inhibiting interactions between the protease and its inhibitors. Moreover, preliminary data suggest increased expression of cathepsin B in the hippocampus and basal ganglia of post-mortem brain tissue from HIV-infected individuals diagnosed with HAND, Alzheimer’s disease, and other neuropsychiatric disorders. These findings provide new evidence for a role of cathepsin B in HIV-1 neuropathogenesis.

## Materials and Methods

### Human Subjects

Research involving human participants was approved by the University of Puerto Rico Institutional Review Board (Protocol 0720109). Blood was collected in ACD tubes for isolation of peripheral blood mononuclear cells (PBMC) after obtaining a written informed consent. Data was analyzed anonymously.

### Isolation and Culture of Primary Macrophages

PBMC were isolated from healthy donors by Ficoll density gradient separation. Adherent monocytes were grown in RPMI supplemented with 20% heat-inactivated FBS, 10% heat-inactivated pooled human sera, and 1% Pen/Strep (all from Sigma Chemical Company, St. Louis, MO) in T25 flasks at a concentration of 1.5×10^6^ cells/ml. Half of the medium was changed every 3 days for all cultures. At seven days, adherent cells were >90% MDM [22].

### Infection of Monocyte Derived Macrophages

HIV-1_ADA_ isolate was kindly provided by Dr. Howard Gendelman (University of Nebraska Medical Center). HIV–1_ADA_ was originally isolated from peripheral blood mononuclear cells of an AIDS patient with Kaposi’s sarcoma and propagated in MDM obtained from HIV-1-seronegative donors after ultracentrifugation as previously described [23]. Viral preparations were screened for endotoxin (10 pg/ml) (Associates of Cape Cod, Woods Hole, Mass.) and Mycoplasma (Gen-Probe II; Gen-Probe, San Diego, Calif.). Viral titer was determined on PHA-blasts as 10^3^ TCID_50_/ml.

After 7 days in culture, MDM were infected with HIV-1_ADA_ at a multiplicity of infection (MOI) of 0.1 or with serum-free media only (uninfected controls) [24]. After overnight incubation, virus was thoroughly washed away and fresh medium was added. Infected MDM were maintained in culture for up to 12 days. Culture supernatants were collected at different days post infection (dpi) depending on the analysis, centrifuged, and stored at −80°C. Infection efficiency was determined in MDM supernatants at 3, 5, 7, 9 and 12 dpi by HIVp24 antigen ELISA, following the manufacturer’s instructions (Express BioTech, Maryland, USA). Protein expression, function, and apoptotic activity were determined in supernatants collected at 3, 6, 9 and 12 dpi. Cells were harvested at the same time points for quantitative intracellular messenger RNA analysis.

### Quantitative Real Time PCR

For quantitative real time PCR experiments, cell pellets were collected at 3, 7, and 12 days post-infection from 8 MDM cultures. RNA was isolated from the pellets using the Qiagen RNeasy Protect kit and quantified using the Nanodrop system. RNA integrity was assessed with the RNA Nanoassay in an Agilent 2100 Bioanalyzer. RNA samples were stored at −80°C for subsequent Real Time PCR analysis. RNA was analyzed for differential expression of cathepsin B, cystatin B, and cystatin C, target genes using GAPH as an internal reference gene, using the Quantitect SYBR Green RT-PCR kit (Qiagen). All primers were tested for their specificity as well as for the absence of primer-dimer formation by PCR, followed by agarose gel electrophoresis. Real time RT-PCR reactions were conducted at a final volume of 25 µL using 20 ng of total RNA and 40 cycles of amplification, as recommended by the Qiagen Handbook, in an ABI StepOne Plus cycler. The fold change of detected amplicons was calculated by comparing the average threshold cycles (Ct) of the reference gene to that of the target genes by the delta delta Ct method [25], [26].

### Preparation of Cell Lysates

Cells were washed in cold PBS and incubated on ice for 30 min with cell lysis buffer (5 mM Tris-HCL buffer at pH 8.0, 0.1% Triton X-100, and protease inhibitor solution, (Sigma), (100 µL/1million cells; Sigma) [cocktail protease inhibitor ratio: 5 µL/100 µL lysis buffer]). Lysates were cleared by centrifugation for 10 min at 1,500 rpm at 4°C, and stored at −80°C for future analyses. Protein concentration was determined using the *DC* protein assay (BioRad, California, USA) following the manufacturer’s instructions.

### Western Blot Analysis

MDM cell lysates containing 30 µg of protein as determined by DC protein assay (BioRad) were subjected to high-speed centrifugation overnight at low temperature to obtain protein pellets. Samples were rehydrated in 12 µl of sample buffer (475 µL of Laemmli sample buffer and 25 µL of β-mercaptoethanol (BME, BioRad) and heated at 70°C for 10 minutes. Samples diluted in sample buffer were loaded onto 4%−20% Tris-HCL 15-well Ready Gels (BioRad), together with a molecular weight marker and positive controls for cystatins B and C (U87 cell lysates) and cathepsin B (isolated human protein from liver, Biovision, California, USA). Gels were run with NuPAGE Protocol at 200 V for 40 min. After electrophoresis, gels were rinsed with PBS and then transferred to a nitrocellulose membrane using the semi-dry transfer method on a transblot apparatus (BioRad) for 30 min (per gel) at 25 V. After transferring, membranes were blocked with 3% BSA in Tween-TBS (Fisher, Philadelphia) for 1 hour at room temperature with shaking. Membranes were probed with rabbit anti-human cathepsin B (1∶500) (Chemicon, Massachusetts, USA); mouse anti-human cystatin C (1∶500); mouse anti-human cystatin B (1∶1000) (Sigma, Missouri, USA), followed by secondary anti-rabbit Ig G- conjugated with Horseradish Peroxidase (HRP) (Sigma) or anti-mouse IgG-HRP Sigma), respectively. All incubations with primary antibodies were done overnight at 4°C, while all incubations with secondary antibodies were done for 1hour at room temperature. Following incubations with primary and secondary antibodies, membranes were washed with TTBS. Chemiluminescence (Super Signal West Femto Detection Kit, Pierce, Massachussets, USA) was used for signal detection. The density of protein bands was determined using the Versa Doc System with Quantity One Software (BioRad) and normalized against the levels of β-tubulin detected with monoclonal mouse anti-β-tubulin (Sigma) as indicated above.

### Cystatins and Cathepsin B ELISAs

Sandwich ELISA (R & D Systems, Minnesota, USA) was used for the quantification of expression of secreted cathepsin B in MDM (1∶100 dilution) following the manufacturer’s instructions and measured in nanograms per milliliter (ng/mL). Extracellular cystatin C was assayed by human cystatin C ELISA (BioVendor, North Carolina, USA) following the manufacturer’s instructions. Briefly, 100 µl of each sample were incubated in a plate pre-coated with polyclonal anti-human cystatin C specific antibody for 30 min. After incubation, the plate was washed and 100 µl of conjugate solution was added to each well and incubated for 30 min. Substrate solution was then added and incubated for 10 min in the dark. The expression of cystatin B was measured with an ELISA (USCN Life Science Inc, China) according to the manufacturer’s instructions. Samples from 5 different donors were tested in duplicates as recommended by the manufacturer. Optical density of all ELISAs was determined by reading absorbance at 450 nm in a Dynex MRX Revelation Microplate Reader (Chantilly, VA).

### Cathepsin B Activity Assay

The bioactivity of secreted cathepsin B was detected using the cathepsin B Activity Assay Kit (Bio Vision, California). This kit is a fluorescence-based assay that utilizes the preferred cathepsin B substrate sequence Arginine-Arginine (RR) labeled with amino-4-trifluoromethyl coumarin (AFC). Samples containing active cathepsin-B cleave the synthetic substrate RR-AFC to release free AFC. The released AFC can easily be quantified by fluorescence. This assay has been used extensively by other researchers to determine active cathepsin B levels [27]. MDM supernatants collected from cell cultures at 3, 6, 9, and 12 days post infection (dpi) were assayed in duplicate following the manufacturer’s instructions. Signal was detected using the VersaFluor TM Fluorometer (BioRad) fluorescence plate reader equipped with a 400-nm excitation filter and a 505-nm emission filter.

The intracellular activity of cathepsin B was determined using the CV-cathepsin B Detection kit (BIOMOL International LP) according to the manufacturer’s protocol. Briefly, PBMCs were cultured in sterile 4-well slide chambers at 2×10^6^ cells per well and allowed to differentiate into MDM for 7 days. At day 7, cultures were inoculated with HIV-1_ADA_ or with serum-free media (uninfected controls) as described above, and kept in culture for 12 dpi. Half of the culture medium was changed every 3 days. At day 12 dpi, arginine-conjugated cresyl violet (CV-RR_2_), was added to the culture media and incubated for 60 min at 37°C in 5% CO2. The attached Arg–Arg group is a preferred substrate for cathepsin B cleavage, and in presence of cathepsin B is detached from CV, which can be detected by its fluorescence when unconjugated. Fluorescence intensity of unconjugated CV was determined at 550 nm for excitation and 610 nm for emission of five fields per well per experimental group. Samples were visualized with a confocal microscope (LSM5 Pascal; Carl Zeiss, Jena, Germany) within 1 hour.

### Neuroblastoma SK-N-SH Cell Cultures

SK-N-SH (HTB-11) cells (human neuroblastoma line, ATCC), were grown and plated on 6-well plates at a density of 7×10^5^ cells per well and maintained in Eagle’s MEM (EMEM) supplemented with 10% fetal bovine serum (FBS; Sigma), 1% sodium pyruvate (Sigma) and 1% non-essential amino acids (Sigma) and incubated for 3–5 days at 37°C, 5% CO_2_ until 70–80% confluence.

### Determination of Cathepsin B Neurotoxic Potential

Confluent SK-N-SH cells were washed twice with PBS and incubated for 24 h with fresh MDM conditioned medium (MCM) from uninfected or HIV-infected MDM cultures derived from four different donors at 1∶4 dilution in plain EMEM. MCM was added with or without a specific cathepsin B inhibitor CA-074 (Sigma) at a concentration of 50 µM. The cathepsin B inhibitor CA074 (L-3-trans-propylcarbamoyloxirane-2-carbonyl)-Lisoleucyl-L-proline) is a very rapid inactivator of cathepsin B with barely detectable action on cathepsins H, L, and S or m-calpain [28]. For experiments examining apoptosis by TUNEL assay and confocal microscopy, SK-N-SH cells were grown and plated on 4-well chamber slides at a density of 1×10^5^ cells per well in the culture media described above. Cells were incubated for 3–5 days at 37°C, 5% CO_2_ until 70–80% confluence. MCM was added to 75–80% confluent SK-N-SH cultures, and incubated at 37°C, 5% CO_2_ for 24 hours. In addition to CA-074, a monoclonal mouse anti-cathepsin B antibody at 1∶500 (Sigma), representing 50x the concentration of secreted cathepsin B, was used to inhibit the enzyme based on the manufacturer’s protocols. The next day, neurons were washed with PBS and fixed using 4% paraformaldehyde. Fixed neurons were incubated for 10 minutes in 3% hydrogen peroxide in methanol to quench auto-fluorescence, and were permeabilized in 0.1%Triton X-100 in 0.1% sodium citrate for 10 minutes on ice. *In situ* TUNEL labeling (ROCHE®) was performed incubating neurons in TUNEL reaction mix (terminal transferase enzyme solution to labeled nucleotides solution ratio 1∶10) for 1 hour at 37°C on a humidity chamber in dark environment. Cells were washed 3 times in PBS, and DAPI, diluted in anti-fade mounting media (Vectashield®) was added to all slides at a final concentration of 2 ng/µL. The negative control consisted of cell incubated in labeling solution without enzyme under the same conditions. The positive control was obtained by incubating fixed and permeabilized cells in 30 U/mL recombinant DNaseI for 10 minutes at room temperature to induce DNA strand breaks, and then labeling them by TUNEL reaction. Confocal microscopy was performed with a Zeiss confocal microscope Axiovert 200 M with a LSM 510 under an excitation wavelength of 488 nm, 20× magnification.

### DuoLink *in situ* Proximity Ligation Assay (PLA) for Protein-protein Interactions

The Duolink II proximity ligation assay kit, composed of anti-rabbit PLA probe plus, anti-mouse PLA probe minus, and detection kit Orange (ex 554, em576) was purchased from Olink Bioscience (Uppsala, Sweden). MDM were cultured in 4 well Premanox chamber slides (Fisher) and inoculated with HIV-1_ADA_ or serum free media (uninfected controls) as described above. At 3, 6 and 12 dpi cells were fixed in 4% paraformaldehyde and permeabilized using 0.1% Triton-X100/BSA and stored in 4% paraformaldehyde solution for future experiments.

Fixed cells were washed with PBS to remove fixing solution and then blocked in a pre-heated humidity chamber with Duolink II Blocking Solution for 30 min at 37°C. Primary antibody mixtures were prepared by diluting antibodies in Duolink II Antibody Diluent at optimal dilutions: cathepsin B (1∶250, Chemicon) cystatin B (1∶1,000, R and D) and cystatin C (1∶250, R and D) and incubated overnight at 4°C with gentle shaking. All Duolink II reagents were diluted according to the manufacturer’s instructions. Samples were air dried and mounted with Duolink II Mounting Media containing DAPI nuclear stain. Detection of the interaction signals was carried out by red fluorescence imaging performed on a Zeiss LSM 5 confocal laser-scanning microscope, equipped with a 63× objective and with an Argon Laser, a 543 He-Ne laser (red), 405 (blue) Laser and a Halogen Lamp.

### Immunofluorescence Staining

To determine the presence of individual proteins in the samples stained for *in situ* PLA, cells fixed in 4% paraformaldehyde were incubated with rabbit-anti-cathepsin B (1∶500, Chemicon); mouse-anti-cystatin B (1∶2,000, R and D) and mouse-anti-cystatin C (1∶500, R and D) and incubated in blocking buffer (3% BSA) overnight at 4°C followed by Alexa-conjugated secondary antibodies anti-rabbit Ig G-543 (1∶500) or anti-mouse IgG-488 (1∶500), Invitrogen). Cells were washed 3–5 times for 10 min with PBS between incubations. Cell preps were allowed to air dry, mounted with Vectashield mounting media (Vector Labs) containing DAPI stain and visualized in a Zeiss LSM 5 confocal laser-scanning microscope as described above.

### Immunohistochemistry of Frozen Human Post-mortem Brain Tissues

Brain tissue samples snap-frozen without cryopreservatives were obtained from the National NeuroAIDS Tissue Consortium, from 7 individuals represented under the following categories: three uninfected, one HIV-infected without cognitive impairment, one HIV-infected with HAD, one HIV-infected with minor cognitive and motor dysfunction (MCMD), one HIV-infected with HIV encephalitis (HIVE) and Alzheimer’s disease, and one HIV-infected with neuropsychological impairment due to other cause (Table 1). For each individual, samples were obtained from three brain regions: basal ganglia, frontal lobe and hippocampus. Samples were fixed in Zambonie solution (2% paraformaldehyde and 0.2% picric acid) for 24 hours at 4°C and then washed with anti-freeze solution (30% Ethylene Glycol, 15% sucrose, 0.1% PBS) and sectioned into 20 µm samples with a microtome using dry ice and 30% sucrose solution. Sections were stored in anti-freeze solution at 4°C, and the original samples were stored in anti-freeze solution at −20°C.

**Table 1:** Post-mortem brain tissues received from the National NeuroAIDS Tissue Consortium.

CASE ID	Path Status	Neurocognitive Status	Age at Death
7102197786	no HIVE^1^	HIV-, no neurocognitive assessment	44
7101267166	no HIVE	HIV-, no neurocognitive assessment	51
7101808383	no HIVE	HIV-, no neurocognitive assessment	51
CE106	no HIVE	Neuropsychological impairment due to other cause: learning disability,probable psychiatric comorbidity (bipolar, schizophrenia)	29
CE168	no HIVE	Normal neurocognition	34
2005	minimal non-diagnostic abnormalities	Subsyndromic neurocognition	44
4028	no HIVE	Possible HAD^2^	46
5007	HIVE, Alzheimer’s	Possible MCMD^3^	37

1)HIVE: HIV encephalitis.

2)HAD: HIV associated dementia.

3)MCMD: Minor cognitive motor disorder.

Sections were washed twice with 1× PBS for 5 minutes, permeabilized with 0.1% Triton X-100 and then blocked using 10% BSA in PBS for 1 hour at room temperature (RT). The following primary antibodies were diluted in blocking buffer and added to the tissue for 12 hours at 4°C: monoclonal mouse anti-cystatin B (R&D systems, 1∶200); polyclonal rabbit anti-human Ionized calcium binding adaptor molecule 1 (Iba1, a specific marker for microglia and macrophages) (Wako, 1∶250); monoclonal mouse anti-human cathepsin B (Sigma, 1∶100). Tissues were washed in PBS 3 times for 5 minutes at RT and. The following Alexa fluor-conjugated secondary antibodies were added: 488 goat anti-mouse and 546 goat anti-rabbit. Secondary antibodies were diluted to 1∶200 in blocking buffer and added for 1 hour in the dark at RT. Tissues were washed 3 times in PBS for 5 minutes in dark environment. Tissue preps were allowed to dry and mounted using Vectashield (VectorLabs®) as anti-fade with or without DAPI nuclear stain (1∶500). Fluorescence was detected using a Zeiss LSM 5 confocal laser-scanning microscope equipped with an argon laser, a 543 He-Ne laser (red), 405 (blue) laser and a halogen lamp, at 63× magnification. For every tissue, two control tissue slides were prepared: one unstained and one stained with secondary antibodies alone. Detector gains in the Pascal Software were manipulated using both types of control tissue preps to minimize unspecific staining and background. Once each color gain was set using the control slides, each slide was examined at least twice and a minimum of two pictures from each sample were taken under the same parameters.

### Analysis

Prior to confirmatory statistical analysis, distributional assumptions were tested using Shapiro-Wilk test of normality. Based on the results of these distributional tests, the assumption of normality could not be supported. Therefore, non-parametric tests statistics were used to address the questions in this research project. Descriptive statistics were calculated for each laboratory measure (e.g. measures of apoptosis), stratified by HIV infection, presence of inhibitor and days post infection, using median and interquartile range (25^th^ and 75^th^ percentile of the distribution). Wilcoxon rank sums were used to test the hypotheses of: 1) no difference between HIV infected and uninfected cultures prior to the introduction of inhibitor; 2) no difference between HIV infected cultures before and after introduction of inhibitor; and 3) no difference between HIV uninfected without inhibitor and HIV infected with inhibitor.

Statistical significance was considered at p<0.05 for all comparisons. All statistics were performed using SAS, version 9.2 (The SAS Institute, Cary, NC).

## Results

### Intracellular Expression of Cathepsin B and its Inhibitors in HIV-1 Infected MDM

**Figure 1:**
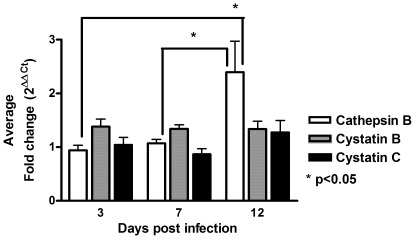
Increased cathepsin B mRNA after HIV infection. MDM from 8 different donors were inoculated with HIV-1_ADA_ or with serum-free media (uninfected controls) for 12 days and cell pellets collected at 3, 7, and 12 days post-infection. Changes in mRNA levels are shown as *Fold change*  = 2 ^ΔΔCt^  = 2 ^(Δ Ct control – Δ Ct experimental)^ for cathepsin B (white), cystatin B (grey), and cystatin C (black). The mRNA levels of cystatins B and C remained similar after HIV infection. A significant increase in mRNA expression was found for cathepsin B in HIV infected MDM at 12 days compared to 3 (*p = 0.038, B) and 7 dpi (*p = 0.028).

It is well known that HIV-1 infection alters host cell biology at both the transcriptional and post-transcriptional level [29], [30]. Previous proteomic studies from our laboratories identified cathepsin B and cystatin B as differentially expressed in HIV-infected macrophages [31]. To determine if HIV-1 has an effect on the expression of genes for cathepsin B and its inhibitors, cystatins B and C, we performed real time PCR of HIV-infected and uninfected MDM cultures from 8 different donors. Samples were analyzed to determine changes in mRNA levels at 3, 7 and 12 dpi. Productive infection was determined in cell supernatants as an increase in HIV p24 protein over the time in culture (data not shown). There was a significant increase in cathepsin B mRNA at day 12 compared to day 3 (p = 0.026) and day 7 post-infection (p = 0.038) (Figure 1). Levels of mRNA for cystatin B and cystatin C did not differ between HIV-1-infected and uninfected samples.

The effect of HIV-1 infection on MDM expression of cathepsin B and its inhibitors, cystatin B and cystatin C, was assessed by Western blot and densitometry analysis. Protein expression was analyzed from 4 donors at 6, 9 and 12 dpi. The relative abundance of intracellular cathepsin B was similar in HIV-1 infected and uninfected control cells (Supplement 1). During peak virus production, cystatin B expression was significantly higher in HIV-infected MDM than in uninfected cells (p = 0.037, Figure S1). We analyzed intracellular expression of cystatin C in MDM after HIV-1 infection and found similar expression in infected and uninfected MDM (Figure S1).

### Cathepsin B is Secreted from MDM at Higher Levels than Cystatin C but is not Higher than Cystatin B

**Figure 2:**
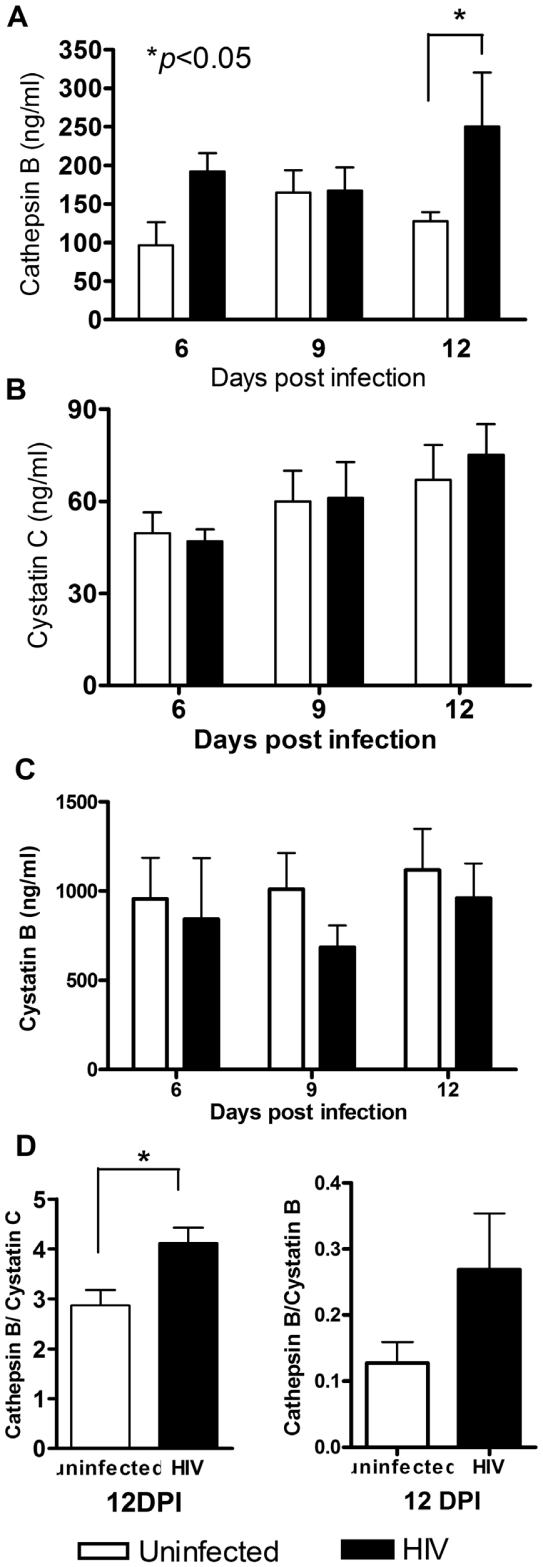
Effect of HIV infection on cathepsin B secretion in macrophages. Cell supernatants (n = 4) from HIV_ADA_-infected (solid bars) and uninfected (open bars) macrophage cultures were collected, centrifuged, and tested for cathepsin B, cystatin B and cystatin C expression by antigen capture ELISA. (A) MDM secreted high levels of cathepsin B at all time points assayed. There was an increase in cathepsin B expression in the HIV-infected samples as compared with uninfected controls at 12 dpi (*p<0.05; A). HIV-infected and uninfected macrophages showed no differences in secretion of cystatin C or B (B and C). The ratio of cathepsin B to cystatin B and cathepsin B to cystatin C were calculated over time in culture (D). Cystatin B was present at higher concentrations than cathepsin B at all time points assayed, as indicated by the ratio of cathepsin B to cystatin B lower than 1. However, an increased cathepsin B to cystatin C ratio was observed in both HIV-infected and uninfected macrophages at all time points. At 12 dpi the ratio of cathepsin B to cystatin C in HIV-infected cells was higher in HIV-infected than uninfected cells (*p<0.05, D).

Under normal conditions, cathepsin B is located inside lysosomes, but oxidative stress induced by HIV-1 infection could stimulate the release of cathepsin B from this cellular compartment. We hypothesized that HIV-1 infection induces the release of cathepsin B from lysosomes to the cytoplasm and the extracellular medium. Therefore, we compared levels of cathepsin B secreted by HIV-infected MDM obtained from 7 different donors with productive infection (as demonstrated by increased levels of HIV-1 p24 antigen over time) to those secreted by MDM obtained from uninfected controls (data not shown). Both uninfected and HIV-infected MDM secreted cathepsin B into the culture medium (range: 125 ng/ml to 590 ng/ml) (Figure 2). However, HIV-infected MDM secreted significantly higher levels of cathepsin B than did uninfected MDM at 12 days post-infection, when virus production and cathepsin B mRNA levels peak (p<0.05). These results suggest that HIV-1 infection induces the synthesis and rapid secretion of cathepsin B into the MDM supernatants.

We next asked if HIV-1 infection could modulate the expression of the cathepsin B inhibitor cystatin C, since the latter is the major extracellular inhibitor of cathepsin B. Expression of cystatin C and the ratio between secreted cathepsin B and cystatin C determines the amount of potentially active cathepsin B in the extracellular medium. We found that the levels of cystatin B and cystatin C in the culture fluids were similar in HIV-1 infected and uninfected MDM throughout the infection (Figure 2B and 2C). We then compared the ratio of secreted cathepsin B to cystatin B and Cystatin C to determine if there was an imbalance between the two proteins that could lead to higher cathepsin B activity. At 12 dpi, cathepsin B was expressed at 2 to 4.5-fold higher levels than cystatin C (p<0.01; Figure 2D). There was a significantly greater increase in cathepsin B relative to cystatin C in HIV-infected versus uninfected MDM (p = 0.030; Figure 2D). These data confirmed that HIV-1 infection induces an imbalance between secreted cathepsin B and cystatin C levels. In contrast, the ratio of cathepsin B to cystatin B revealed that cathepsin B is expressed at significantly lower levels than cystatin B (Figure 2D).

### HIV-1 Infection Increases Secreted Cathepsin B Activity in MDM

**Figure 3:**
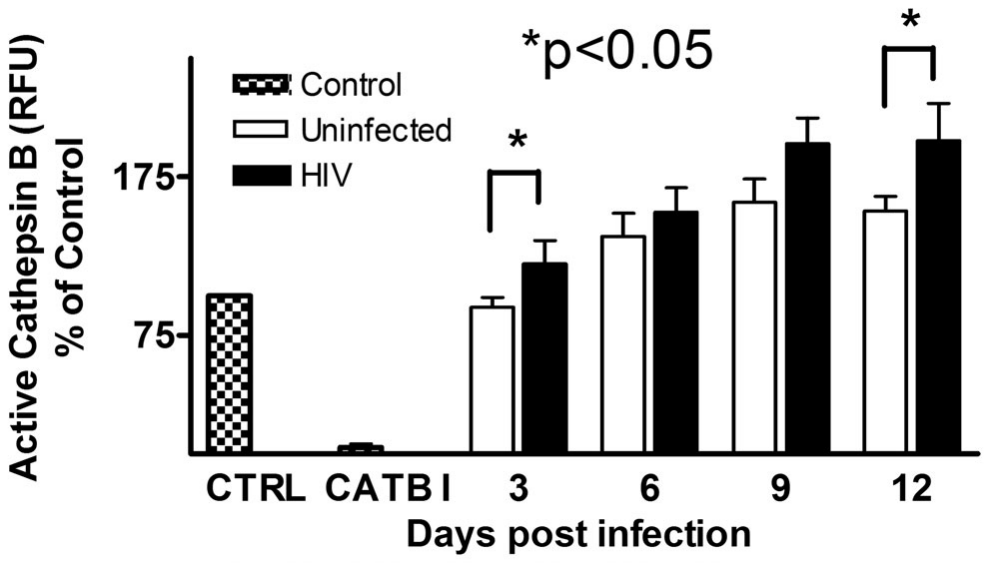
Secreted cathepsin B is more active in HIV-infected macrophages than in uninfected controls. Protein activity was measured by adding a synthetic peptide specific for cathepsin B conjugated to a red fluorogenic compound (RR_2_-AFC) and read in a fluorometer at 400 nm excitation and 505 nm emission filters. Culture fluids from HIV-infected macrophages showed a significant increase in the activity of secreted cathepsin B at 3 and 12 days post infection (*p≤0.05). Results for cathepsin B activity are expressed as percentages of control (media only). Increased cathepsin B activity over the days after HIV infection (mean estimate increase/day 9.25 (SE 2.61), p = 0.002). The specificity of the assay is shown by the abrogation of any active cathepsin B after the addition of an inhibitor.

To determine the activity of intracellular cathepsin B in HIV-infected and uninfected samples, we isolated and cultured MDM from four additional female donors for *in vitro* infections with HIV-_ADA_. Cell lysates and culture fluids were collected at 3, 6, 9 and 12 dpi. There was a significant increase in HIV p24 antigen during the time after 6 days post-infection (data not shown). Cathepsin B intracellular activity in both HIV-infected and control uninfected samples remained unchanged throughout the infection (Figure 3). To determine the extracellular activity of cathepsin B, supernatants from MDM cultures were assayed for cathepsin B activity using a fluorescently-labeled cathepsin B substrate. Fluorescence intensity reflected cathepsin B activity, which was expressed as the percentage of a negative control (MDM culture media). Both HIV-infected and uninfected cultures secreted active cathepsin B (Figure 3). However, there was a significant increase in cathepsin B activity in HIV-infected MDM relative to uninfected MDM over time in culture (p = 0.002) with a mean estimated increase of 9.25±2.61 RFU/day. Cathepsin B activity was significantly increased at 3 and 12 days post-infection (p = 0.048 and p = 0.043, respectively, Figure 3). A trend of increased cathepsin B activity was observed at days 6 and 9 post-infection. These results indicate that MDM normally secrete active cathepsin B, and that cathepsin B secretion is increased at 12 days post-infection, when viral production peaks. Hence, these results provide preliminary evidence for a correlation between viral production and cathepsin B activity, although further validation of this correlation with a larger number of donors will be required.

### MDM-secreted Cathepsin B Contributes to Neuronal Apoptosis Induced by HIV-1 Infection

**Figure 4:**
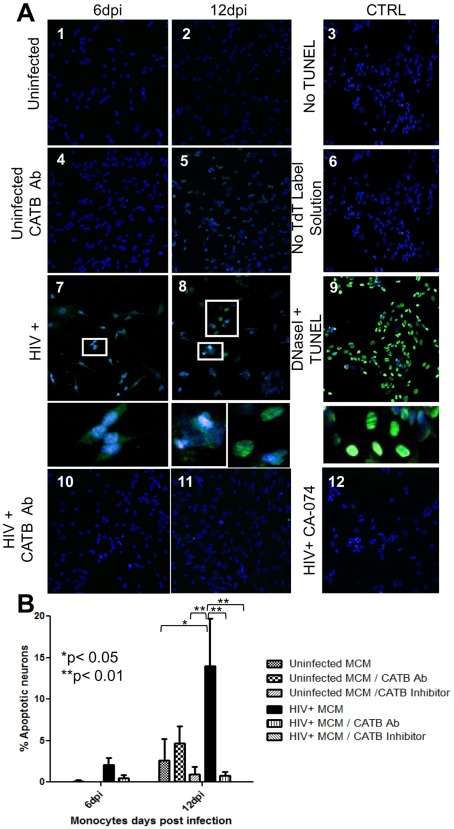
Cathepsin B contributes to neuronal apoptosis caused by HIV-infected MDM. Apoptosis was measured using terminal deoxynucleotidyl transferase dUTP nick end labeling (TUNEL) assay at 6 and 12 days post-infection as shown by green fluorescence in neurons stained with DAPI (blue) (Panel A). These results in panel A are representative of four experiments. The SK-N-SH cells exposed to MCM from uninfected MDM (1–2) with the cathepsin B antibody (3–4) do not show apoptosis. Neuronal apoptosis increased after exposure to HIV infected MCM (5–6) from later times post- infection. Inhibition of cathepsin B with the specific inhibitor CA-074 decreased the neuronal apoptosis in each of the time points (7–8). Pretreatment of medium with a monoclonal antibody against cathepsin B at 1∶500 dilution also decreased neuronal apoptosis (9–10). Quantitative analysis of staining ratio of apoptotic (green )/non-apoptotic (blue) nuclei using Image-based Tool for Counting Nuclei (ITNC) from Image J software (NIH) revealed a significant increment in percentage of apoptotic neurons at 12 dpi (p<0.05) compared to neurons treated with uninfected macrophage conditioned media. However, inhibition of cathepsin B by CA-074 decreased significantly the percentage of apoptotic neurons at 12 dpi (p<0.01) compared with neurons treated with HIV-infected media. MCM supernatant pre-treated with cathepsin B antibody reverted the percentage of apoptotic neurons at 12 dpi (p<0.01). Results in panel B represent the mean +/− SD of four biological replicates.

To determine the role in neuronal injury of HIV-induced cathepsin B secretion by MDM, differentiated neuronal cells from the neuroblastoma cell line SK-N-SH were incubated with MCM from four uninfected and HIV-infected cultures collected at 6 and 12 dpi. For this experiment, we determined the optimal concentration of MCM by comparing undiluted and 1∶2 or 1∶4 diluted MCM. The 1∶4 dilution was chosen for all subsequent experiments (data not shown). Neuronal apoptosis was determined using TUNEL labeling and the images were analyzed by confocal microscopy (Figure 4A). A significant increase in percentage of apoptotic neurons was observed after exposure to MCM obtained at 12 days post-infection with HIV-1 (p<0.05) compared to neurons treated with uninfected MCM. The apoptosis induced by MCM obtained at 12 dpi was significantly decreased (p<0.01) by addition of the specific cathepsin B inhibitor CA-074 to the medium. A similar decrease in apoptotic activity was seen when the MCM was pre-treated with cathepsin B antibody (Figure 4B) (p<0.01). Results are represented as mean +/− SD of four different experiments.

### HIV-1 Infection Induces the Release of Cathepsin B from Lysosomes

**Figure 5:**
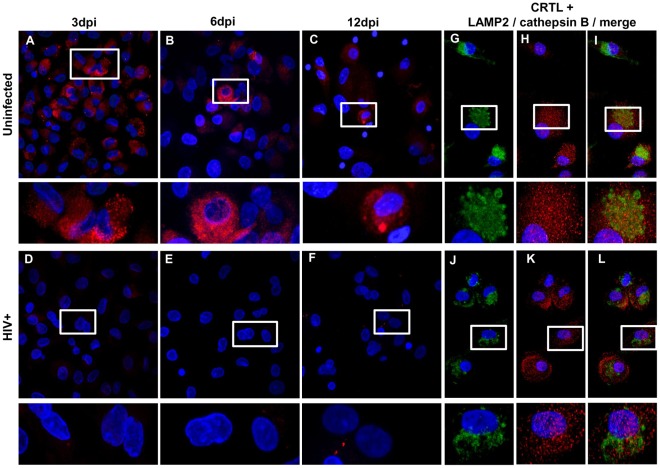
Cathepsin B is released from lysosomes in HIV-infected MDM. To analyze the lysosomal localization of cathepsin B, cathepsin B and LAMP2 immunoreactivity were assessed by *in situ* PLA (Duolink) in uninfected and HIV-infected MDM 3, 6 and 12 dpi. Cathepsin B colocalizes with LAMP2 in uninfected MDM (A, B and C; top panels). However, little colocalization is seen in HIV infected MDM (D, E and F; bottom panels). The presence of individual proteins was determined by immunofluorescence staining (G, H, I, J, K and L). As seen in the right panels both, cathepsin B (red) and LAMP2 (green) are expressed in uninfected (G, H, and I) and HIV-infected (J, K, L) cells. The results presented in this figure are representative of 3 experiments.

Cathepsin B is a protease of lysosomal origin [32] whose secretion is preceded by translocation from the lysosome to the cytoplasm. We hypothesized that HIV-1 infection induces the release of cathepsin B from the lysosome to the cytosol and subsequently to the extracellular space. To visualize cathepsin B within lysosomes before and after HIV-1 infection, we performed double-immunofluorescence studies of cathepsin B and the lysosomal-associated membrane protein 2 (LAMP2) by *in situ* PLA co-localization assay (Figure 5). Cathepsin B localized to lysosomes is shown as red fluorescent signal, which that is detectable only if the two cathepsin B and LAMP2 are present in close proximity. The results showed little or no red fluorescence in HIV-infected samples (Figure 5D, E and F, bottom panel) compared to uninfected controls (Figure 5A, B and C, top panel) at all time points assayed. Thus, cathepsin B disappeared from lysosomes after HIV-1 infection. We confirmed that the absence of red fluorescence was due to absence of cathepsin B from lysosomes by immunofluorescent staining of cathepsin B and LAMP2 with Alexa-conjugated secondary antibodies (Figure 5, right panel). Both LAMP2 (green) and cathepsin B (red) were expressed at high levels in uninfected controls (Figure 5G, H and I) and HIV-infected samples (Figure 5J, K and L). The merged image of LAMP2 and cathepsin B staining shows little or no co-localization of the two proteins in the HIV-infected samples (Figure 5L). This data indicates decreased levels of cathepsin B within lysosomes in HIV-infected samples, suggesting that cathepsin B is released to other compartments.

### HIV-1 Inhibits the Interactions between Cathepsin B and its Inhibitors

**Figure 6:**
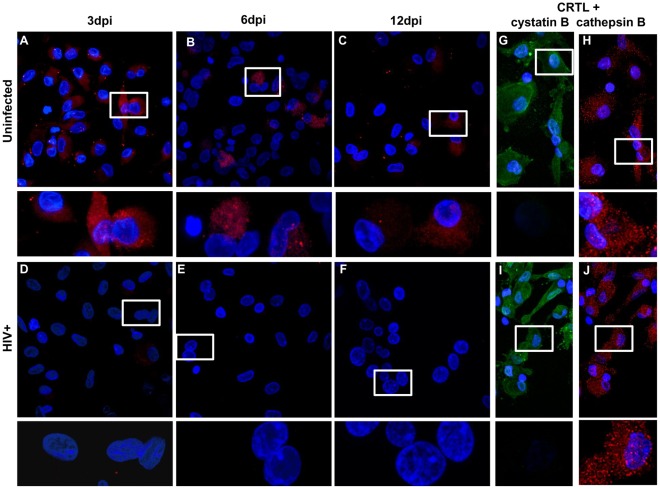
Cathepsin B does not interact with cystatin B in HIV-infected MDM. *In situ* PLA (Duolink) assay showed interaction between cathepsin B and cystatin B in uninfected (A, B and C; top panels) and decreased interactions in HIV infected MDM (D, E and F; bottom panels). Expression of cathepsin B (red) and cystatin B (green) was confirmed by immunofluorescence in uninfected (G and H; right top panels) and HIV-infected MDM (I and J; right bottom panels). This is a representative figure from 3 experiments performed.

**Figure 7:**
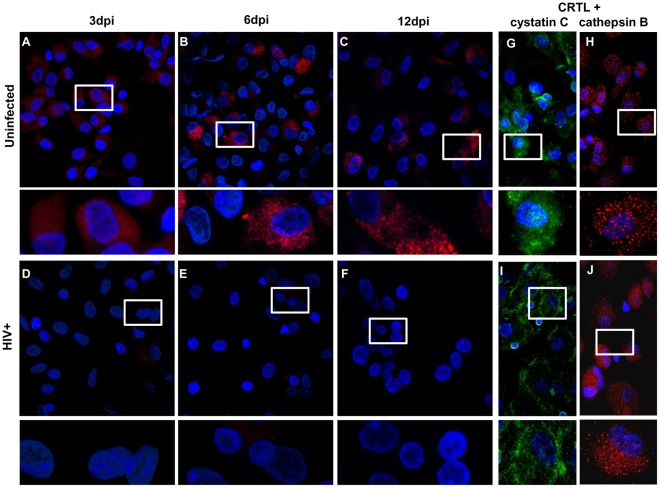
Cathepsin B does not interacts with cystatin C in HIV-infected MDM. *In situ* PLA (Duolink) assay shows interaction between cathepsin B and cystatin C in uninfected cells (A, B and C; top panels) and decreased interactions in HIV infected MDM (D, E and F; bottom panels). Expression of cathepsin B (red) and cystatin C (green) was confirmed by immunofluorescence in uninfected (G and H; right top panels) and HIV-infected MDM (I and J; right bottom panels). This is a representative figure from 3 experiments performed.

To further understand the role of cathepsin B inhibitors in cathepsin B secretion and neurotoxicity after HIV-1 infection, we analyzed the protein-protein interactions between cathepsin B and cystatins B and C by *in situ* PLA (Figures 6 and 7). The presence of individual proteins was confirmed by immunofluorescent staining (Figures 6 and 7, left panel). As expected, results from uninfected controls showed that cathepsin B interacts with both cystatin B (Figure 6, top panel) and cystatin C (Figure 7, top panels) at all time points assayed. However, little or no interaction between cathepsin B and its inhibitors was seen in HIV-1-infected samples (Figure 6 and 7 bottom panels). These results provide strong evidence that HIV-1 infection not only induces the secretion of bioactive cathepsin B, but also inhibits and the protease’s interactions with its inhibitors. This dysfunction in protease/inhibitor interactions might facilitate the secretion of bioactive cathepsin B.

### Cathepsin B and Cystatin B Expression in Brains of HIV-infected Individuals with Cognitive Impairment

**Figure 8:**
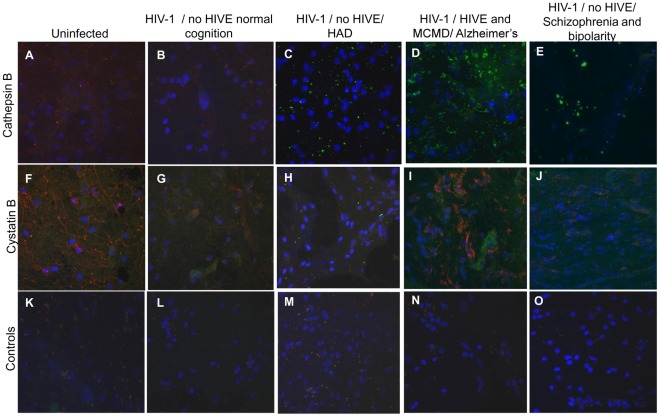
Expression of cathepsin B and cystatin B in the hippocampus of post-mortem brain tissues. Hippocampal tissue from HIV-seronegative (A, F and K) and HIV-seropositive (B-E, G-J and L-O) patients were stained with mouse anti-human cathepsin B followed by Alexa 488 conjugate goat anti-mouse (green), or mouse anti-human cystatin B followed by Alexa 488 conjugate goat anti-mouse (green), and rabbit anti-human Iba-1 followed by Alexa 546 goat anti-rabbit (red), and nuclear staining by DAPI (blue). Hippocampus samples from the same patients stained only with secondary antibodies and DAPI were used as negative controls illustrated in K-O. For each color, detector gains were maintained standard in every caption taken with the Pascal software in a Zeiss LSM 5 confocal laser-scanning microscope using a 63× magnification.

We did a preliminary analysis of cathepsin B and cystatin B expression in samples of post-mortem brain tissue obtained from three uninfected and four HIV-infected individuals. Cathepsin B protein was undetectable in hippocampus of HIV-negative individuals (Figure 8A) and the HIV-positive individual with normal cognition (Figure 8B). In contrast, low levels of cathepsin B were seen in the hippocampus of an HIV-1 positive individual with HIV-associated dementia (HAD) (Figure 8C), and higher levels in an individual with mild cognitive motor disorder (MCMD) (Figure 8D). We should emphasize that the latter individual had two additional neurological complications: HIV encephalitis (HIVE) and Alzheimer’s disease. In addition, low levels of cathepsin B staining were seen in the hippocampus of an individual with a history of neuropsychological impairment due to schizophrenia and bipolar disorder (Figure 8E). Interestingly, however, cystatin B as well as cathepsin B immunoreactivity was elevated in the hippocampus from the HIV-1 positive individual with MCMD (Figure 8I). Double-staining of tissue with an antibody to the macrophage marker Iba-1 did not significant overlap with cathepsin B or cystatin B staining, suggesting that latter proteins are localized either extracellularly or in other cell populations. Future experiments will be conducted with antibodies against neurons, astrocytes, and vascular endothelial and smooth muscle cells to further explore the identities of the cell populations expressing these two enzymes in HIV-infected brains. Hippocampus samples from the same patients stained only with secondary antibodies and DAPI were used as negative controls and did not show immunoreactivity (Figures 8K–O).

**Figure 9:**
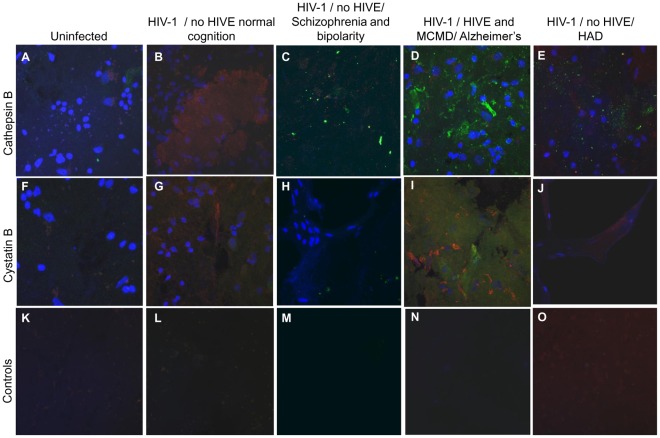
Expression of cathepsin B and cystatin B in the basal ganglia of post-mortem brain tissues. Basal ganglia tissue from HIV-seronegative (A, F and K) and HIV seropositive (B-E, G-J and L-O) patients were stained with mouse anti-human cathepsin B followed by Alexa 488 conjugate goat anti-mouse (green), or mouse anti-human cystatin B followed by Alexa 488 conjugate goat anti-mouse (green), and rabbit anti-human Iba-1 followed by Alexa 546 goat anti-rabbit (red), and nuclear staining by DAPI (blue). Unstained tissues were used as controls as illustrated in K to O. Magnification: 63×.

Results similar to those seen for hippocampus were obtained in the basal ganglia (Figure 9). In contrast, no changes in cathepsin B or cystatin B immunoreactivity were observed in frontal lobe tissue samples from HIV-infected individuals relative to controls (data not shown).

The number of post-mortem brain samples that could be obtained for this analysis were small, and the tissue was not optimally fixed and preserved for immunocytochemistry. Thus, further study of cathepsin B and cystatin B expression *in vivo* will be necessary. Nonetheless, these results suggest that cathepsin B is upregulated in the brains of HIV-infected individuals with cognitive impairment.

## Discussion

Infiltrating macrophages and resident microglia are the principal producers of HIV-1 in the CNS, and the major contributors to viral neuropathogenesis [33]. Proteomic analyses of HIV-infected macrophages revealed that HIV-1 infection induces profound alterations in the normal physiology of macrophages, which could contribute to neuronal dysfunction [31]. These changes include not only the production of neurotoxins, but also the dysregulation of normal cellular processes. We used macrophages differentiated from blood monocytes from healthy donors to explore cellular mechanisms of neuronal apoptosis in the brain after HIV-infection. Our study sought to determine whether HIV-1 infection could impact the interplay between cathepsin B and its inhibitors in macrophages. In the present study, we found that HIV-1 infection modulates the expression, secretion and activity of cathepsin B and of its natural inhibitors, cystatins B and C. We also found that secreted bioactive cathepsin B contributes to neuronal apoptosis, which can be reversed by the addition of a specific cathepsin B inhibitor or an antibody to cathepsin B. This data suggested a dysregulation of cathepsin B compartmentalization and inhibition systems. Our results demonstrated that cathepsin B disappears from lysosomes after HIV-1 infection, suggesting its release from the organelle. This phenomenon occurred in parallel with the disappearance of the interactions between cathepsin B and both its inhibitors that were seen in uninfected control cells. To our knowledge, this is the first study that links macrophage-secreted cathepsin B with the neuronal apoptosis associated with HIV-1 infection.

Many studies have consistently linked the presence of infected and highly activated MP with the onset of early signs of neuronal injury [34]. These cells are important sources of inflammatory molecules and neurotoxic products such as TNF-α [35], IL-1β and IL-6 [35] NO [36], glutamate [37], platelet activating factor [38], quinolonic acid [39], [40], arachidonic acid [41], and viral proteins (e.g., gp120 and Tat). In many cases, secretion of toxic products by macrophages occurs as a consequence of profound physiological alterations caused by HIV-1, [22], [42], [43], [44], [45] and in turn alters altering the cells’ phenotype and ultimately their protective functions.

Proteomic analyses have enabled the identification of hundreds of proteins that are differentially or uniquely expressed in HIV-1 infected cells compared to uninfected cells [31]. Cathepsin B and other proteins belonging to the same papain-like cysteine protease family, as well as their inhibitors have been identified in HIV infected macrophages by several research groups [43], [46], [47], [48].

To determine whether cathepsin B might play a role in the neuronal injury induced by HIV, we first studied the effect of HIV-1 infection on gene and protein expression. We found increased transcription of cathepsin B in HIV- infected MDM after 12 dpi, when HIV replication peaked. However, intracellular cathepsin B expression remained constant throughout infection in cultured macrophages, suggesting increased secretion of the enzyme. We confirmed that HIV-infected macrophages secreted highly toxic levels of cathepsin B (250 ng/ml; 10 µM; Figure 3B) compared to uninfected cells. These results are consistent with other studies using microglia stimulated with chromogranin A, where concentrations of 10 µM (100 ng/ml) cathepsin B induced significant neuronal apoptosis [19]. Although HIV-1 infection did not affect intracellular cathepsin B levels *per se,* it increased the enzyme’s secretion and activity in HIV-infected MDM relative to uninfected control cells at 3 and 12 dpi. However, we also found unexpectedly high levels of secreted cathepsin B in uninfected cells, which suggests that these cells could be secreting cathepsin B mostly in its precursor forms: i.e., as part of normal cathepsin B trafficking mechanisms (as reviewed by Brix and collaborators [32]). It remains to be determined if HIV-1 infection causes increased processing of cathepsin to the functional forms.

Intracellular expression of cystatin B was also modulated by HIV-1 infection in macrophages. With time in culture, the expression of cystatin B increased in HIV-1 infected macrophages and reached significantly higher levels than those seen in uninfected cells. These results are consistent with previous observations made in our laboratory, where Luciano-Montalvo et al. [49] found increased cystatin B expression in MDM infected with another macrophage-tropic HIV-1 strain (HIV-1_Bal_) after 12 dpi. However, increased cystatin B protein with no differences in mRNA levels may reflect intracellular retention of this enzyme, as demonstrated by a tendency to achieve lower levels of secretion in HIV-infected cultures as compared to uninfected controls.

Cysteine proteases, including cathepsin B, are ubiquitous host proteins involved mainly in non-selective intracellular protein degradation in lysosomes [50]. Outside lysosomes, cathepsins are tightly regulated by cystatins [50]. [51]. Until recently, cathepsins were thought to be completely inactive at neutral pH, but several groups have provided evidence associating cytoplasmic and secreted cathepsin B with inflammation [52], [53], [54] and apoptosis [19], [21], [55], [56], [57], [58]. It is well known that HIV infection triggers TNF- α and IL-1 inflammatory pathways [28] and that action is associated with increased oxidative stress [21], [50] and antioxidant dysfunction [45] during HAND. Oxidative stress [59] and TNF-α [60] can promote the release of cathepsin B from lysosomes. Our results indicate a significant decrease in the interactions between cathepsin B and the lysosome in HIV-1 infected MDM, suggesting that HIV-1 triggers the release of cathepsin B from this organelle. Similar results found by others associated the translocation of cathepsin B from the lysosome to the cytosol with apoptosis in other inflammatory diseases [61], [62]. Consistent with a possible link between oxidative stress and cathepsin B activity, recent studies by several groups showed that antioxidants can prevent lysosomal damage and subsequent cathepsin B release and activity. For example, treatment with proanthoyanidin antioxidants, which are members of the flavonoid family, resulted in a decrease in oxidative stress and levels of lysosomal enzymes, including cathepsin B [63]. Furthermore, addition of gallic or caffeic acid prevented lysosomal damage and reduced levels of cathepsin B activity, respectively [63], [64]. These studies confirm that cathepsin B can be released from lysosomes in response to oxidative stress, and suggest the potential use of antioxidants as therapeutic agents to prevent cathepsin B release and neurotoxicity.

Another potential mechanism whereby HIV might affect cathepsin activity is through increased lysosomal permeability. The HIV protein Nef has been shown to directly promote lysosomal membrane permeabilization, with resulting efflux of cathepsins into the cytosol [65]. An alternative or additional mechanism would involve generalized activation of proteasomal proteins, and there is evidence that another viral protein, Vif, activates the proteasome pathway to target antiviral proteins for degradation as a mechanism to enhance viral infectivity [66]. As cathepsins are the main proteins involved in proteasomal degradation, increased activity of this pathway triggered by Vif might promote the synthesis of cathepsin B, which could result in its over-expression and accumulation. Finally, it has been shown that HIV infection can induce the translocation of cystatin B from the cytosol to the plasma membrane [67]. This sequestration of cystatin B to the membrane limits the availability of this protein in the cytoplasm to inhibit released cathepsin B. Thus, multiple alterations in macrophage physiology induced by HIV-1 infection may act together to affect cathepsin B availability and activity.

When levels of cathepsin B released from lysosomes exceed those of available cystatins, the protease inhibitor ratio is disrupted, and free active cathepsin B can indiscriminately degrade essential proteins and/or be secreted from the cell [50]. In this study, we showed that MDM released active cathepsin B and that HIV-1 infection increased levels of active cathepsin B over time of infection. Other groups have associated increased cathepsin B activity with infections by several other viruses, such as human Papilloma virus [68], Influenza A virus [69], Adeno-associated virus [70], and Norovirus [71]. In terms of cathepsin B’s mechanism of action after viral infection, our observations confirm the results of Furman’s group, and extend them by showing that not only does cathepsin B increase after viral infection, but the activity of secreted cathepsin B is also modulated.

At low concentrations, cytoplasmic cathepsin B can be modulated by cystatins. Cystatins are the endogenous inhibitors of cysteine proteases, with cystatin B and cystatin C being the two major inhibitors of cathepsin B. It was believed that cystatin B acted primarily in the intracellular compartment, while cystatin C was actively secreted to act on extracellular cathepsin B. However, recent studies on HIV and other inflammatory diseases have shown high levels of secreted cystatin B in response to either HIV infection or inflammation [43], [49]. Our results are consistent with these findings, as we showed an increase in cystatin B secretion in response to HIV-1 infection. Cystatin C has also been found by others to respond to HIV and other viral infections [46], [72]. However, we found no differences in the levels of secreted cystatin C after HIV-1 infection. Changes in the expression of the two principal inhibitors of cathepsin B, cystatin B and cystatin C, could also represent a redundant mechanism to prevent damage caused by free cathepsin B. However, an imbalance in the expression levels of these two proteins could lead to an increase in free active cathepsin B, which in turn could lead to neuronal dysfunction during HAND. Our findings suggest that, although intracellular cystatin B expression increases after HIV infection in MDM, neither cystatin B or cystatin C inhibits cathepsin B activity.

Imbalance between cathepsin B and its inhibitors has been reported in other inflammatory conditions such as pelvic inflammatory disease [54] and broncopulmonary dysplasia [73] In both of those studies, cathepsin B was expressed at higher levels than its inhibitors and thus contributed significantly to cell damage. In this study, we analyzed the ratio of secreted cathepsin B to cystatin C in culture supernatants after HIV infection of MDM. We found that cathepsin B levels were 2 to 4.5-fold higher than cystatin C levels at all times, with a significant increase in the cathepsin B/cystatin C ratio in HIV-infected cells. An imbalance in the cathepsin B/cystatin C ratio implies the possibility of a dysfunction in the interactions between the cystatins and cathepsin B. Our data demonstrates that cathepsin B interacts with its inhibitor in uninfected MDM, however in HIV-infected MDM there is little or no interaction between cathepsin B and either cystatin B or C. This indicates that HIV-1 not only modulates the expression of cathepsin B but it also inhibits protease: inhibitor interactions, promoting in consequence an increased active cathepsin B secretion. This dysfunction might allow the release of active cathepsin B into the extracellular space, which could then promote neuronal apoptosis.

An important goal of our study was to determine if cathepsin B could have a potential role in HIV neuropathogenesis by analyzing its intracellular and extracellular expression and activity in MDM relative to HIV-1 infection. We studied neuronal apoptosis using the neuroblastoma cell line SK-N-SK, which has been used to study pathways of neuronal apoptosis in several neurodegenerative diseases, including HIV-1 associated neurodegeneration [74], [75], [76]. Although we did not induce the cells to differentiate with retinoic acid, nonetheless the cells expressed heavy neurofilament protein, a marker used to identify mature neurons (Figure S2). Our results provide evidence for the first time of a role for MDM-secreted cathepsin B in the neuronal apoptosis induced by HIV-1 infection. Specifically, we showed that by inhibiting the high levels of cathepsin B secreted by HIV-infected MDM, the neurotoxic activity of supernatants from these cells could be abolished.

Cathepsin B has been associated with apoptosis, by both caspase-dependent and independent pathways. It has been shown that release of cathepsin B from lysosomes after TNF-α treatment enhances mitochondrial release of cytochrome c and subsequent caspase activation. In this model, deletion of cathepsin B gene resulted in diminished apoptosis [19]. Several groups have shown that the most likely route of cathepsin B-induced apoptosis is through the cleavage of the Bcl-2 family pro-apoptotic member, Bid [53], [77], [78]. However, Houseweart and collaborators reported that cathepsin B promotes apoptosis in absence of the pro-apoptotic protein Bid [79]. These observations suggest that cathepsin B mediates apoptosis by multiple pathways and that in the absence of Bid, other molecules can substitute for its pro-apoptotic role. Thus, targeted inhibition of extracellular cathepsin B could represent a useful addition to the therapeutic strategy for HAND patients. Future studies will address if other cathepsins also contribute to the neurotoxic activity of HIV-infected MDM, as has been shown in other inflammatory diseases [80].

To further explore the possible role of cathepsin B and cystatin B play in HAND, we did immunohistochemical analyses of post-mortem tissue from three brain regions of uninfected and HIV-1 positive individuals: hippocampus, basal ganglia, and frontal lobe. Cathepsin B and cystatin B proteins showed increased expression in the hippocampus and basal ganglia of HIV-infected individuals with MCMD and HAD compared to that seen in those brain regions in the uninfected individual and an HIV-infected individual with normal cognition. These results are consistent with previous observations of increased lysosomal enzymes in post-mortem brain tissue with HIV encephalitis [81], and provide further support for our hypothesis that cathepsin B is involved in HAND. Previous studies also demonstrated that perivascular macrophages, microglia, and astrocytes have increased lysosomal activity in the white matter during HAD [81]. Other studies demonstrated that there is high activation of macrophages and microglia in the basal ganglia and hippocampus of HIV-infected people, despite suppression of HIV RNA in plasma due to HAART therapy [82]. Therefore, these brain regions are susceptible to an immune reaction during late stages of the infection.

Increased cathepsin B was observed in the three HIV-infected individuals with Alzheimer’s disease. Alzheimer’s disease is characterized by abnormal accumulation of certain brain proteins, including β-amyloid and tau [83]. Cathepsin B functions as a β-secretase in the production of β-amyloid peptides [84], and human cystatins B and C have the ability to directly bind to β-amyloid peptides [85], [86], [87], [88]. Skerget and coworkers found that cystatin B prevents β-amyloid fibril formation *in vitro* [89]. In another study in an Alzheimer’s disease mouse model, small-molecule inhibitors of cathepsin B decreased β-amyloid levels and improved memory performance [90]. These studies, together with our results, raise the possibility that HIV-associated increases in cathepsin B levels contribute to the development of Alzheimer’s disease in HIV-infected individuals.

In conclusion, our results demonstrate that HIV infection dysregulates cathepsin B in macrophages, at both the mRNA and protein levels, leading to increased secretion of bioactive cathepsin B that contributes to neuronal apoptosis. In addition, our data shows a failure of cystatins B and C to prevent the secretion and activity of cathepsin B in the extracellular environment, probably due to dysfunction in their interactions with cathepsin B. Future experiments will be aimed at determining what protein modifications in cystatin B prevent this interaction. In addition, further study of cathepsin B expression in the brains of HIV-infected individuals with and without cognitive impairment will be necessary to confirm the role of cathepsin B in HAND.

## Supporting Information

Figure S1 **Intracellular expression of cathepsin B and its inhibitors after HIV infection in macrophages.** HIV replication was measured in the cell supernatants by p24 viral antigen ELISA (A). *In vitro* HIV-infected macrophages from 4 different donors (solid circles) showed an increase in HIV replication at 12 dpi (p<0.001; A). Differences in expression of cathepsin B, cystatin B, and cystatin C at 6, 9 and 12 dpi; panels B, C and D and E) were determined by Western blot analysis. Aliquots of 30 µg of total protein were loaded per line blotted and probed with antibodies against cathepsin B, cystatin B, cystatin C (B). The density of the bands was normalized (C, D and E) against that of β-tubulin (A bottom panel). Normalized data are presented for cathepsin B (open bars), cystatin B (grey bars), and cystatin C (solid bars). Levels of cathepsin B in HIV-infected and uninfected cells remained unchanged through infection (C, D and E). Cystatin B levels were significantly higher (*p<0.05) in HIV-infected MDM compared with uninfected controls at 12 dpi (p≤0.05; E). No changes were seen in the levels of cystatin C during HIV infection (C-E).
(TIF)

Figure S2 **Neurofilament staining of SK-N-SH cells in culture.** SK-N-SH neuroblastoma cells were cultivated in slide chambers, and fixed with a methanol/acetone solution. Primary antibody MAB 5266 MS x Neurofilament 200 kD (Chemicon Temecula, CA) was used to stain heavy neurofilaments at 1∶1000 dilution followed by 1 hr incubation at room temperature. A secondary antibody (Alexa 488 Goat Anti-Mouse IgG) was added at 1∶2000 dilution and incubated for 1 hour at room temperature. DAPI was used for nuclear staining (blue). Panels A to E represent different fields to demonstrate that SK-N-SH show evidence of maturation by positive neurofilament staining. Confocal images were obtained on a Zeiss confocal microscope Axiovert 200 M with a LSM 510 with 63× magnification (panels A and B) with a 2.5 zoom amplification (panels C to E).
(TIF)
