# Supplementary figures and images for: Dysregulation of Macrophage-Secreted Cathepsin B Contributes to HIV-1-Linked Neuronal Apoptosis
Source: PLoS One. 2012 May 31;7(5):e36571. doi: 10.1371/journal.pone.0036571 (PMC3365072; doi:10.1371/journal.pone.0036571)

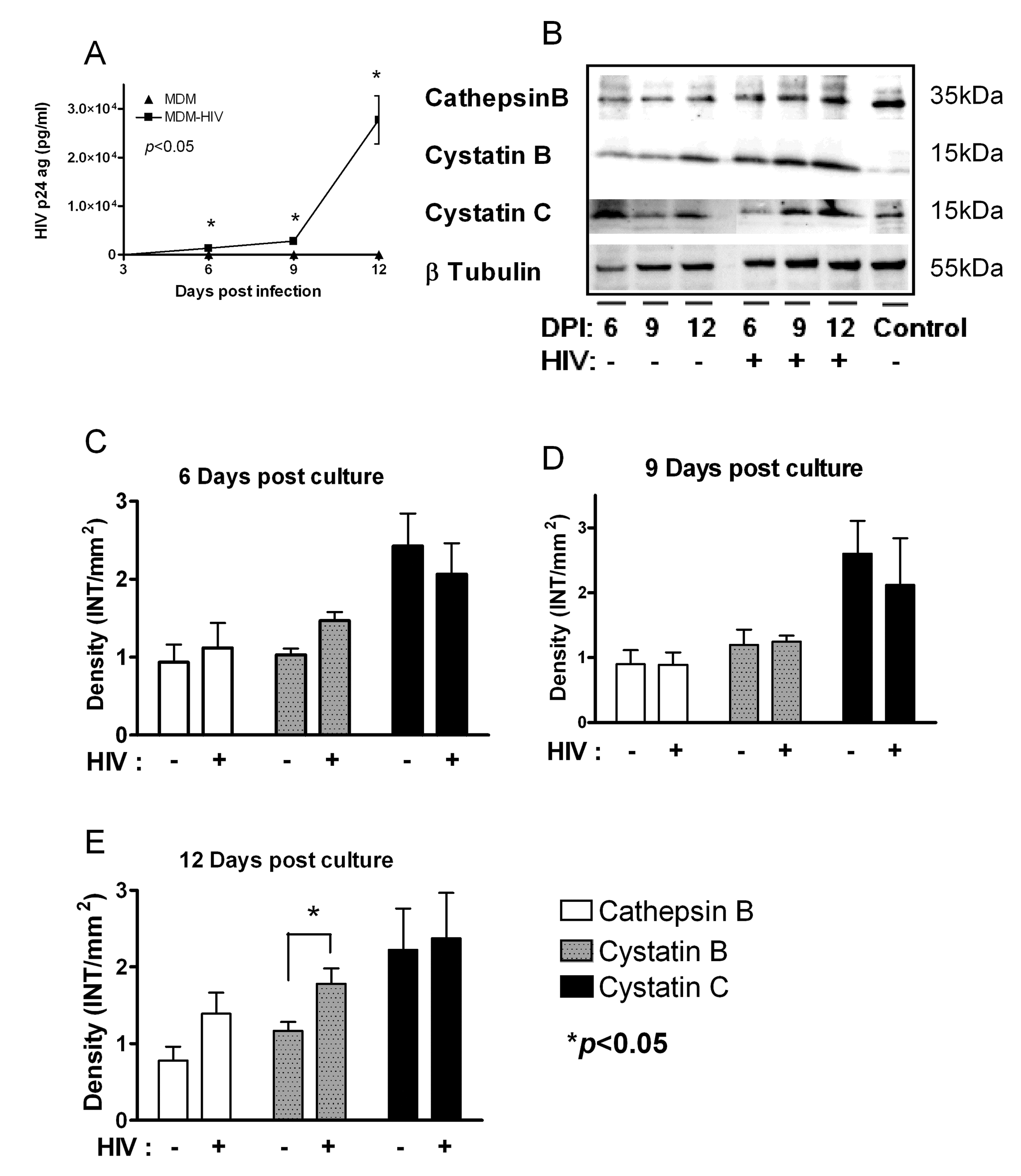

Supplement: Figure S1 — Intracellular expression of cathepsin B and its inhibitors after HIV infection in macrophages. HIV replication was measured in the cell supernatants by p24 viral antigen ELISA (A). In vitro HIV-infected macrophages from 4 different donors (solid circles) showed an increase in HIV replication at 12 dpi (p<0.001; A). Differences in expression of cathepsin B, cystatin B, and cystatin C at 6, 9 and 12 dpi; panels B, C and D and E) were determined by Western blot analysis. Aliquots of 30 µg of total protein were loaded per line blotted and probed with antibodies against cathepsin B, cystatin B, cystatin C (B). The density of the bands was normalized (C, D and E) against that of β-tubulin (A bottom panel). Normalized data are presented for cathepsin B (open bars), cystatin B (grey bars), and cystatin C (solid bars). Levels of cathepsin B in HIV-infected and uninfected cells remained unchanged through infection (C, D and E). Cystatin B levels were significantly higher (*p<0.05) in HIV-infected MDM compared with uninfected controls at 12 dpi (p≤0.05; E). No changes were seen in the levels of cystatin C during HIV infection (C-E). (TIF) [file pone.0036571.s001.tif]

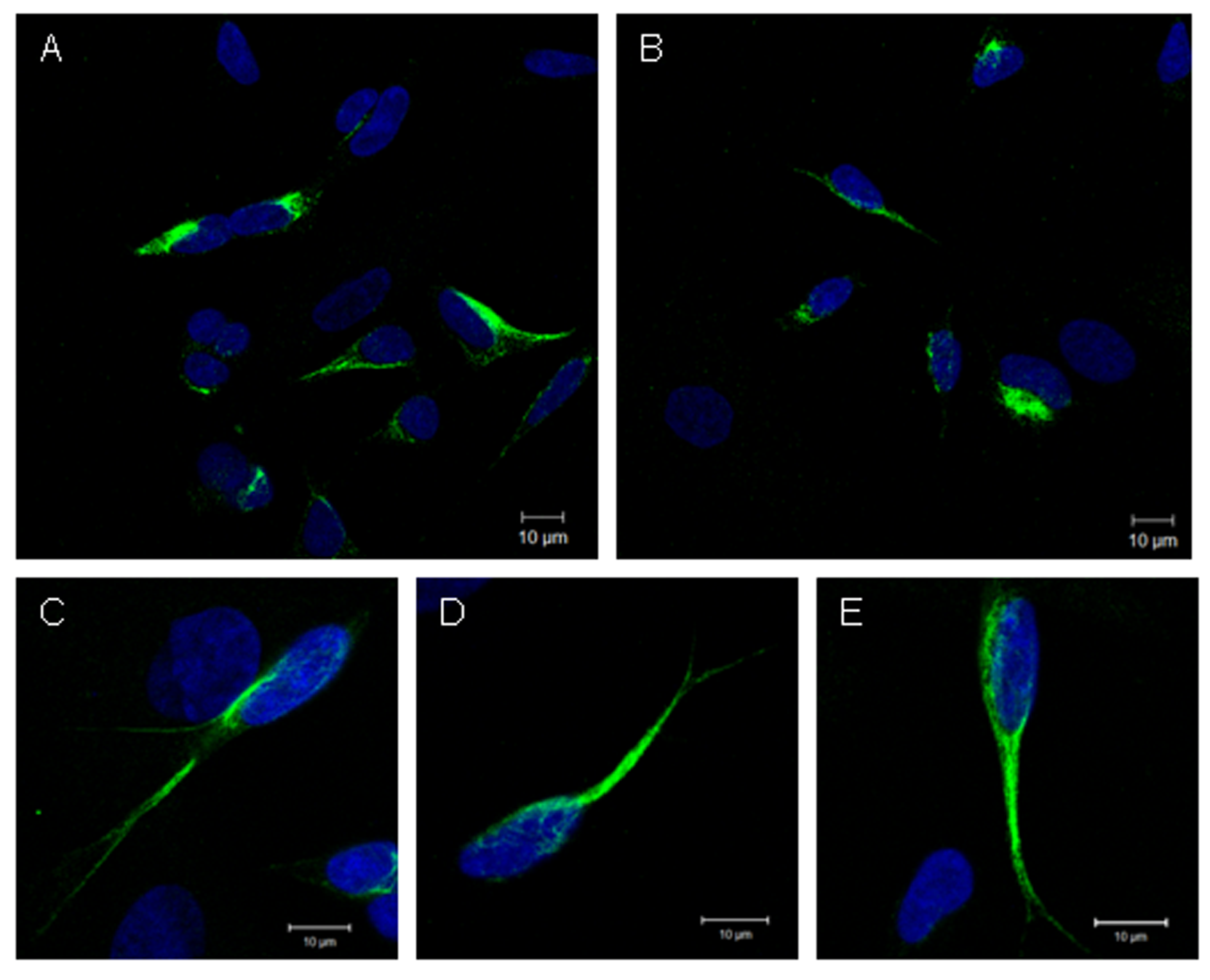

Supplement: Figure S2 — Neurofilament staining of SK-N-SH cells in culture. SK-N-SH neuroblastoma cells were cultivated in slide chambers, and fixed with a methanol/acetone solution. Primary antibody MAB 5266 MS x Neurofilament 200 kD (Chemicon Temecula, CA) was used to stain heavy neurofilaments at 1∶1000 dilution followed by 1 hr incubation at room temperature. A secondary antibody (Alexa 488 Goat Anti-Mouse IgG) was added at 1∶2000 dilution and incubated for 1 hour at room temperature. DAPI was used for nuclear staining (blue). Panels A to E represent different fields to demonstrate that SK-N-SH show evidence of maturation by positive neurofilament staining. Confocal images were obtained on a Zeiss confocal microscope Axiovert 200 M with a LSM 510 with 63× magnification (panels A and B) with a 2.5 zoom amplification (panels C to E). (TIF) [file pone.0036571.s002.tif]
